# Human Herpesvirus and the Immune Checkpoint PD-1/PD-L1 Pathway: Disorders and Strategies for Survival

**DOI:** 10.3390/microorganisms9040778

**Published:** 2021-04-08

**Authors:** Takayuki Murata

**Affiliations:** Department of Virology and Parasitology, Fujita Health University School of Medicine, Toyoake 470-1192, Japan; tmurata@fujita-hu.ac.jp

**Keywords:** HSV, VZV, CMV, EBV, KSHV, PD-1, PD-L1

## Abstract

The immune system has evolved as a complex and efficient means of coping with extrinsic materials, such as pathogens and toxins, as well as intrinsic abnormalities, such as cancers. Although rapid and timely activation of the immune system is obviously important, regulated downregulation of the system is almost as significant as activation to prevent runaway immunity, such as allergies and hypercytokinemia. Therefore, the immune checkpoint programmed cell death 1 (PD-1)/programmed cell death ligand 1 (PD-L1) pathway is beneficial for the host. On the other hand, pathogens have evolved to evade host immunity by taking advantage of the PD-1/PD-L1 pathway. This review is focused on human herpesviruses, such as herpes simplex virus (HSV), cytomegalovirus (CMV), and Epstein–Barr virus (EBV), which cause various types of disorders, and their relationships with the PD-1/PD-L1 pathway. Understanding such relationships will be useful for developing preventative and therapeutic methods for disorders caused by herpesviruses.

## 1. Introduction

Herpesviruses are large, enveloped viruses, with a large DNA genome contained within an icosahedral nucleocapsid. One of the best-known characteristics of herpesviruses is that they can establish lifetime latent infection in addition to lytic infection. During latency, herpesviruses remain silent, expressing only a subset of viral genes, and are hidden from host immunity. However, these viruses occasionally reactivate from latency into an active mode, in which almost all viral genes are expressed; viral DNA replication takes place; and, eventually, progeny virus particles are produced [1,2].

The family of Herpesviridae is divided into three subfamilies: α-, β-, and γ-herpesviruses [3]. The α-herpesviruses typically grow faster than the members of the other subfamilies and, thereby, cause acute infectious illness. These viruses infect a relatively broad range of cells, including skin, mucosal, lymphoid, liver, spleen, neuronal, endothelial, and fibroblast cells, and establish latency in nerve ganglia. There are three human α-herpesviruses: herpes simplex virus 1 (HSV-1), HSV-2, and varicella-zoster virus (VZV). HSVs cause herpes labialis, genital herpes, and herpes encephalitis, while VZV is responsible for chickenpox and shingles [4].

The β-herpesviruses have the largest genome (200–250 kb) among the Herpesviridae, and they have slower growth than the members of the other subfamilies. These viruses target epithelial, mucosal, lymphoid, liver, spleen, neuronal, endothelial, and fibroblast cells, especially under conditions where the host immune system is weak. They establish latency in myeloid and lymphoid cells, and/or their precursors. Cytomegalovirus (CMV) is a member of the human β-herpesviruses, which causes interstitial pneumonia, retinitis, and congenital cytomegalovirus infection, and has also been implicated in glioblastoma. Others include human herpesvirus 6A (HHV-6A), HHV-6B, and HHV-7, the latter two of which are causative agents of exanthema subitem in infants [5].

Epstein–Barr virus (EBV) and Kaposi sarcoma-associated herpesvirus (KSHV) are two members of the human γ-herpesviruses. Both viruses predominantly infect B cells, in which they occasionally establish latent infection. They can also infect other cell types, such as epithelial, mucosal, lymphoid, liver, spleen, endothelial, and fibroblast cells. It must be emphasized that these γ-herpesviruses have oncogenic properties. For example, EBV is associated with Burkitt’s lymphoma (BL), Hodgkin’s lymphoma (HL), post-transplant lymphoproliferative disorder (PTLD), diffuse large B cell lymphoma (DLBCL), natural killer (NK)/T cell lymphoma (NKTCL), gastric carcinoma (GC), and nasopharyngeal carcinoma (NPC), and KSHV is related to Kaposi sarcoma, Castleman disease, and primary effusion lymphoma [6].

Programmed cell death 1 (PD-1) is a type I membrane protein, initially identified as a gene involved in programmed cell death. PD-1 is a member of the immunoglobulin superfamily and is expressed as a monomer on the cell surface of not only activated T cells but also other immune cells, including B cells, NK cells, dendritic cells, and monocytes. The interaction of programmed cell death ligand 1 (PD-L1) with PD-1 activates the Src homology region 2-containing protein tyrosine phosphatase 2 (SHP2) signaling pathway. SHP2 downregulates signaling molecules in the T cell receptor pathway, such as Zeta-associated protein of 70 kDa, phosphatidylinositol 3-kinase, and phospholipase Cγ2, and suppresses T cell activation. Fine-tuning of immunity by the PD-1/PD-L1 checkpoint provides advantages for the survival of the host, because an excessively strong immune response for a long period will have adverse health effects [7].

**Figure 1:**
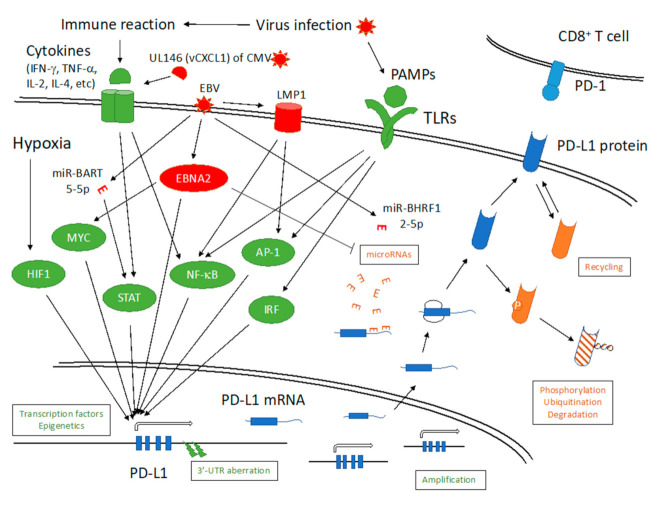
Schematic diagram of regulation of programmed cell death ligand 1 (PD-L1) gene expression. Green color indicates positive regulators, while orange denotes negative regulators. Red color indicates viral factors. IFN-γ, interferon-γ; TNF-α, tumor necrosis factor-α; IL-2, interleukin-2, CMV, cytomegalovirus; UL146, unique long 146; vCXCL1, viral chemokine (C-X-C motif) ligand 1; EBV, Epstein–Barr virus; LMP1, latent membrane protein 1; PAMPs, pathogen-associated molecular patterns; TLRs, toll-like receptors; HIF1, hypoxia-inducible factor 1; MYC, myelocytomatosis oncogene; STAT, signal transducer and activator of transcription; NF-κB, nuclear factor-κB; AP-1, activator protein 1; IRF, interferon regulatory factor, miR-BHRF1-2-5p, microRNA-*Bam*HI H fragment rightward frame 1-2-5p.

PD-L1 is expressed in hematopoietic cells, as well as endothelial, epithelial, fibroblast, and nerve cells. Cytokines, such as tumor necrosis factor-α(TNF-α), interferon-γ (IFN-γ), and interleukin 4 (IL-4), upregulate expression of PD-L1 through signal transducer and activator of transcription (STAT) and nuclear factor-κB (NF-κB) pathways and induce exhaustion of CD8^+^ T cell-mediated immunity (Figure 1). Transcription factors, such as hypoxia-inducible factor 1, myelocytomatosis (MYC), activator protein 1 (AP-1), and interferon regulatory factor (IRF), have been reported to play positive roles in PD-L1 expression. PD-L1 expression can also be increased by chromosomal abnormalities, such as translocation and focal amplification. Fusion of the *PD-L1* locus with transcriptionally active gene loci may occur by translocation, leading to induction of *PD-L1* gene expression. Focal amplification involves increased copy number of the region including *PD-L1*. In addition, microRNAs (miRNAs), posttranslational modifications, degradation, and transportation of the protein play regulatory roles in its surface expression. When cancer cells overexpress PD-L1, the cells can elude CD8^+^ T cell-mediated cell killing because PD-L1 causes exhaustion of T cells by activating SHP2 signaling. This is one of the mechanisms by which cancer cells become resistant to tumor immunity. On the other hand, administration of PD-1/PD-L1 inhibitor can reactivate CD8^+^ T cell-mediated tumor immunity. Indeed, inhibitory antibodies against PD-1 or PD-L1 have shown excellent clinical effects in at least some types of cancer [8,9,10,11].

Similar to the way in which cancer cells evade host tumor immunity by taking advantage of the PD-1/PD-L1 immune checkpoint, viruses also exploit this pathway. PD-L1 expression is induced by infection with influenza virus, human immunodeficiency virus, adenovirus, and Ebola virus [12,13]. As herpesviruses have complicated lifecycles and induce diverse symptoms and diseases, ranging from acute infection and inflammatory disorders to cancers, the mechanisms by which they evade the immune system are complex. This review article presents an overview of the relationships between each human herpesvirus and the PD-1/PD-L1 pathway that have been demonstrated in previous studies.

## 2. HSV

The relationship between HSV and the PD-1/PD-L1 pathway was first reported in the herpetic stromal keratitis mouse model in which ocular HSV-1 infection was shown to induce PD-1 and PD-L1 expression in T cells and macrophages, respectively, in the cornea and lymph nodes, and administration of an antagonistic antibody to PD-L1 aggravated keratitis [14]. These findings suggested that symptoms of herpetic stromal keratitis are exacerbated when the immune response is strong, and the PD-1/PD-L1 pathway ameliorates the disease by restraining the immune response. However, other reports indicated that HSV infection causes upregulation of PD-1/PD-L1, and increased PD-1/PD-L1 results in exhaustion of cell-mediated immunity, thereby increasing viral copy number. For example, blockade of PD-L1 by monoclonal antibody restored CD8^+^ T cell function in the ganglia in a mouse model of latent infection [15]. The latency-associated transcript of HSV-1 was also implicated in induction of PD-1/PD-L1 in the trigeminal ganglia tissue in a mouse model of latent infection, which was associated with exhaustion of CD8^+^ T cells and increased virus copy number [16,17]. PD-L1 levels were increased by HSV-1 infection in dendritic cells [18,19] and epithelial cells of the cornea [20]. It has been speculated that PD-L1 induction may be mediated by inflammatory cytokines induced by virus infection, and pathogen-associated molecular patterns (PAMPs) may also be involved in induction of PD-L1 expression via Toll-like receptors (Figure 1). 

In addition, HSV has been used as an oncolytic virus, and use of PD-1/PD-L1 pathway inhibitors in combination with oncolytic HSV was shown to result in increased antitumor immunity [21,22,23].

## 3. VZV

VZV infection causes dysregulation of PD-L1 levels in a cell type-dependent manner and was shown to downregulate PD-L1 protein expression in many types of cells, including fibroblasts [24]. As *PD-L1* mRNA levels are not altered by VZV infection, the observed decrease in PD-L1 protein level is likely to be attributable to posttranscriptional regulation. The reduced level of PD-L1 expression by VZV resulted in higher levels of inflammation, which could contribute to the development of VZV-associated vasculopathy. On the other hand, peripheral blood mononuclear cells (PBMCs) expressed elevated levels of PD-L1 when infected with VZV and blocking of PD-L1 resulted in increased CD8^+^ T cell-mediated immunity [25]. These observations suggest that inhibition of the PD-1/PD-L1 pathway may enable efficient clearance of the virus in chickenpox and shingles. Other groups also reported that increased PD-L1 expression in macrophages resulted in impaired T cell activation and expansion, leading to a defective VZV-specific T cell immune response [26].

## 4. CMV

On infection with murine CMV, dendritic cells of mice showed elevated levels of PD-L1 expression, resulting in tolerance or anergy in antigen-specific T cells [27]. Antibody-mediated blockade of the PD-1/PD-L1 pathway led to increased immune reaction in viremic CMV patients after renal transplantation [28]. A recent report [29] indicated that the unique long 146 (UL146) gene of CMV, which encodes a viral homolog of viral chemokine (C-X-C motif) ligand 1, augmented PD-L1 expression at both mRNA and protein levels in hepatocyte cell cultures through activation of STAT3 (Figure 1). Knockdown of PD-L1 reduced the effects of UL146 and upregulation of CD8^+^ T cell immunity. This may not be the only mechanism of PD-L1 induction by CMV, but the observation that a viral gene is involved in evading host immunity by upregulating PD-L1 expression is of interest. In addition, CMV has been shown to be associated with glioblastoma and, therefore, CMV-specific adoptive T cell therapy is currently being evaluated. Interestingly, the levels of PD-L1 expression in tumor cells were lower or even undetectable in glioblastoma patients who exhibited longer survival following T cell therapy against CMV, suggesting that inhibition of the PD-1/PD-L1 pathway may be a promising treatment strategy, especially when combined with CMV-specific adoptive T cell therapy in glioblastoma patients.

## 5. HHV-6A, 6B, and 7

Infection of primary monocytes with HHV-6B upregulated surface expression of PD-L1, which was likely to have been meditated by activation of reactive oxygen species (ROS) and STAT1/3 pathways [30]. Therefore, HHV-6/7 may also exploit the immune checkpoint pathway for survival.

## 6. EBV

As EBV has oncogenic properties, the relationships of the PD-1/PD-L1 pathway and EBV or EBV-associated cancers have been studied extensively. Indeed, PD-1 and PD-L1 are expressed in many EBV-positive cancers, such as HL, PTLD, DLBCL, NPC, GC, and NKTCL, as well as chronic active Epstein–Barr virus infection (CAEBV), and in infiltrating immune cells [31,32,33,34,35,36,37,38,39,40,41]. PD-1/PD-L1 inhibitor products have already been approved or shown to have marked efficacy for treatment of several cancers, including HL, NKTCL, NPC, and GC [42,43,44,45,46,47]. EBV induces PD-L1 expression through multiple mechanisms (Figure 1). In NPC cells, EBV drives the expression of latent membrane protein 1 (LMP1) and IFN-γ, which in turn activate STAT, AP-1, and NF-κB pathways and induce PD-L1 [33]. Similarly, LMP1 activates AP-1 and NF-κB pathways and increases PD-L1 in NKTCL [48]. EBV nuclear antigen 2 (EBNA2) has also been linked with PD-L1 induction in B cells. It was reported that EBNA2 downregulated miR-34a through EBF-1 and induced PD-L1 in BL and DLBCL [49]. Our group presented evidence that EBNA2 acted as a transcriptional coactivator for PD-L1 by binding to enhancer sequences [50]. It is also possible that EBNA2 increases PD-L1 via induction of MYC gene expression [50]. In addition to miR-34a, a number of host-encoded miRNAs, such as miR-15, 200, and 513, play roles in regulation of PD-L1 [10]. It should also be noted that cancers, including those positive for EBV, frequently have aberrations in the 3′-untranslated region (UTR) of the PD-L1 gene in the genome [36,38,39,51,52]. This indicates that regulation of PD-L1 by miRNAs is very strong, and cells that acquire resistance to these miRNAs could evade host immunity by increasing PD-L1 expression and proliferate as cancers. Interestingly, EBV also encodes an miRNA (miR-BHRF1-2-5p) that negatively regulates PD-L1 expression through binding to the 3′-UTR of PD-L1 mRNA [53]. On the other hand, another study demonstrated that EBV miR-BART-5-5p downregulated PIAS3, a negative regulator of STAT, and thereby upregulated PD-L1 expression in GC [54]. These reports indicate that EBV has acquired many mechanisms of activating the PD-1/PD-L1 pathway for its survival.

## 7. KSHV

Another member of the γ-herpesvirus subfamily, KSHV, is also associated with some cancers, but its relationship with the PD-1/PD-L1 pathway has not been studied as extensively as EBV. There have been some reports of PD-L1-positive cancers that were infected with both EBV and KSHV [55,56]. Strong expression of PD-1 and PD-L1 were reported in KS specimens from AIDS patients [57]. In addition to cancers, KSHV can infect monocytes and macrophages and was shown to induce expression of PD-L1 [58,59]. Interestingly, lytic KSHV infection has been shown to increase PD-L1 expression [57,59]. Although the specific lytic gene of KSHV responsible for PD-L1 induction has not been identified, proinflammatory cytokines induced by lytic infection may in turn induce PD-L1, likely through NF-κB, STAT, and AP-1 signaling pathways [57,59].

## 8. Conclusions

Infection by herpesviruses can trigger an inflammatory response in the host, resulting in increased local cytokine/chemokine levels. Such cytokines and chemokines can induce expression of PD-1 and PD-L1, not only in infected cells but also in the surrounding cells through activation of NF-κB, STAT, IRF, and AP-1 signals. This is part of the host mechanism to restrain the runaway immunity, but if the degree of constraint is too strong, the host will not be able to kill the pathogen. Therefore, the host cells have established sophisticated systems to fine tune the PD-L1 level, such as miRNAs that play a role in regulating PD-L1 overexpression. Interestingly, EBV has already developed a countermeasure to the miRNA to evade immunity in that EBNA2 decreases miR-34a expression. Currently, only limited data are available regarding the precise molecular mechanisms by which herpesviruses manipulate the PD-1/PD-L1 pathway, but such mechanisms may be encoded by herpesviruses other than EBV because herpesviruses have many such genes and noncoding RNAs. Further studies are required, therefore, to elucidate these issues.
